# Exploring the acceptability, barriers, and facilitators to psychosis screening in the integrated behavioral health primary care setting: a qualitative study

**DOI:** 10.1186/s12913-024-11359-4

**Published:** 2024-08-13

**Authors:** Mark Savill, Rachel L Loewy, Sarah Gobrial, Julianna Kirkpatrick, A. Jonathan Porteus, Tyler A Lesh, J. Daniel Ragland, Tara A Niendam, Cameron S Carter

**Affiliations:** 1 University of California Davis, Sacramento, USA; 2 University of California San Francisco, San Francisco, USA; 3 WellSpace Health, Sacramento, USA

**Keywords:** Prodromal questionnaire – brief, Schizophrenia, Clinical high-risk syndrome, Primary care, Pathways to care, Screening, Qualitative interviews

## Abstract

**Background:**

A longer duration of untreated psychosis (DUP) is associated with poorer treatment outcomes. Screening for psychosis spectrum disorders in the primary care setting could help support the earlier detection and treatment of individuals in need. However, the acceptability of screening for psychosis in this setting as part of routine care is currently unknown.

**Methods:**

We conducted a qualitative interview study with providers and service users who participated in an early psychosis screening program conducted in an integrated behavioral health primary care (IBH-PC) setting. Interviews were recruited from one of eight WellSpace Federally Qualified Health Center IBH-PC clinics in the Sacramento, CA area. Transcripts of the recorded interviews were analyzed using thematic analysis.

**Results:**

In total, 12 providers and eight service users participated in the interviews. Most service user and provider participants were supportive of psychosis screening in an IBH-PC setting, but not as part of the general practitioner consultation due to the brief, non-behavioral health nature of many of the appointments, and the expected low prevalence of psychosis in this population. The support of leadership, adequate training and support, staff turnover, and organizational changes were all seen to impact the successful implementation of the program. Different barriers and facilitators were considered important at each stage of the process from introducing the screening procedures to service users; to determining when, where, and how to screen; and how to effectively manage the referral and post-referral stages.

**Conclusions:**

Despite the additional challenges of screening in an IBH-PC setting relative to secondary mental health services, the process was considered acceptable and feasible to providers and service users. Services that plan to conduct psychosis screening in their clinics need to consider the challenges and their potential solutions to implementation at each stage of the screening process.

**Supplementary Information:**

The online version contains supplementary material available at 10.1186/s12913-024-11359-4.

## Introduction

A short duration of untreated psychosis (DUP) is considered critical in early psychosis (EP) care given its impact on treatment response [1, 2]. However, the median DUP in the USA is 1–3 years [3], far exceeding the World Health Organization recommendations [4]. Consequently, developing strategies to reduce DUP represents an important avenue to improving EP care outcomes.

DUP comprises of two components; the time it takes for the individual to encounter health services subsequent to the onset of their first psychotic episode (“demand-side” DUP), and the subsequent time it takes to navigate the care pathway to appropriate treatment (“supply-side” DUP) [5, 6]. “Supply-side” DUP typically comprises a greater proportion of the overall DUP length [7], highlighting the importance of reducing the time taken to navigate to EP care. One strategy includes screening service users in settings such as community mental health clinics (CMHCs). To date, these campaigns in both Europe and the US have been relatively successful, finding a higher number of service users eligible for EP services relative to usual care [8, 9], while being acceptable and feasible to integrate into standard practice [10].

While psychosis screening in CMHCs may represent one strategy to increase the identification of those who may benefit from specialized early psychosis care, implementing psychosis screening even earlier in the care pathway – namely primary care (PC) – could yield even greater benefits. In the UK and Canada, the PC general practitioner (GP) has been found to be amongst the most frequently reported first healthcare contacts on the pathway to treatment for psychosis [6, 11, 12], despite referrals from PC to EP services being rare [13, 14]. Furthermore, evidence suggests individuals in contact with PC providers may have longer DUP relative to those who do not [13], which may be attributable to the under-identification of less prominent psychotic symptoms in this setting [15, 16]. Consequently, interventions in the PC space have been considered a key avenue to improving the identification and care engagement among individuals experiencing psychosis spectrum disorders [17].

Population-based screening requires relatively little training and specialty knowledge to implement and has been accepted by service users in PC for conditions such as depression and substance use disorder [18, 19]. However, additional challenges to screening in CMHCs relative to PC include the much shorter appointment times, which makes implementing even brief screening interventions challenging [20]; and the low prevalence and broader case mix of service users, which can reduce screening accuracy [21, 22]. One way to mitigate these challenges could be to conduct screening in integrated behavioral health (IBH) departments within PC services. In recent years the integration of these services has undergone expansion across the US, supported by the widespread adoption of the patient-centered medical home model [23]. The IBH clinics are typically co-located with the GP clinic, thus reducing the risk of losing service users as they navigate through services. However, as a behavioral health clinic, they are likely to have a higher prevalence of psychosis cases and a less broad case mix of service users, both of which may improve the acceptability to providers and service users, and the accuracy of the screening tool. Finally, while the median length of routine GP appointments in the US is only 15 min [24], appointments in IBH settings are typically longer (i.e., 30–50 min), meaning more time for screening may be available.

In a recent study by our group conducted in the US, screening for psychosis spectrum disorders in an IBH-PC setting was found as an effective method to identify individuals with psychosis spectrum disorders, identifying individuals that may otherwise have been missed [25]. However, this was completed within the context of a research study, and it is unclear how feasible or acceptable such an approach may be in routine clinical care. Consequently, in this study, we conducted an exploration of the acceptability, and barriers and facilitators to the implementation of routine population screening for EP in an IBH-PC setting from the perspective of service users and providers. Additionally, participants discussed the viability of extending such a process into the GP appointment to determine if this represents an acceptable strategy for identifying service users even earlier in the care pathway.

## Methods

### Design

A semi-structured qualitative interview study was conducted in 2019–2020 to explore service user and provider experiences of completing a screener for EP in an IBH-PC setting. The screening procedure included service users completing a tablet-based version of the Prodromal Questionnaire – Brief [26], either before, during, or after their intake assessment. The research was conducted utilizing a constructivist approach [27, 28], exploring people’s knowledge regarding the screening process, and if and how it should be implemented in this setting, through their lens of their own experiences.

### Setting

The study took place across eight participating WellSpace Health PC health centers across the Sacramento region. All participating clinics are federally qualified health centers (FQHC), meaning the clinics provide services irrespective of an individual’s ability to pay. Consequently, these clinics typically serve individuals who are historically underserved. At each clinic, physical and behavioral health services are co-located to support care integration. Service users are typically referred from the GP clinic to the IBH clinic either based on clinical judgment or a positive Patient Health Questionnaire-9 (PHQ-9) screen [29]. Referrals to IBH services are facilitated by care coordinators who support both physical and behavioral health providers and service users.

### Participants

Participants included service users and providers who had experienced either delivering or completing the screening procedure at one of the participating WellSpace clinics. All service user participants aged 18–30 attending a WellSpace IBH intake appointment were eligible to participate in the screening study. All service users who participated in the screening study were then eligible to take part in the current interview study. The only additional exclusion criterion was an inability to speak English at a level that precluded participation in the interview. All providers that either assisted in the administration of the screening procedure or managed those that completed the procedures, were eligible to participate. For both service user and provider participants purposive sampling was employed. For service users, this meant individuals that screened positively and negatively, and those that were found to be eligible and not eligible for specialty early psychosis services at the phone screen stage were actively sought. Individuals that screened positively were oversampled given 1) those that were screened positively and referred to a program would have more experiences to share, relative to those that just completed a 21-item screener in the IBH-PC setting, and 2) following a positive screen more outcomes were possible, and it was considered necessary to represent each (i.e., a false positive screen, screening identifying a person with psychosis spectrum disorder leader to the offer of specialized care, and screening identifying a person with psychosis spectrum disorder not meeting criteria for services). This was considered particularly important given the possibility that people may have different perspectives of the process depending upon the outcome.. For the provider sample care coordinators, licensed clinicians, and senior management were purposively recruited to ensure that all aspects of the implementation of the screening procedure were considered.

### Procedures and data analysis

Detailed procedures for the screening process are reported elsewhere [25]. Before conducting the interviews, topic guides for service user and provider participants were developed (see supplementary materials) and amended following review by WellSpace staff. Approximately nine months into the screening procedure WellSpace leadership at each screening site was contacted about the study via email, who in turn notified their staff. Interested provider participants either contacted research staff via email to express interest in participating or else met with the research team during a site visit. For service user participants, WellSpace providers informed service users about the study, and with their agreement research staff contacted them to further discuss the study. Interviewers introduced themselves as researchers tasked with improving the implementation of the screener, and the interview as an opportunity to better understand what was working well, what was not working well, and if they considered screening for psychosis appropriate in this setting.

Interviews were hosted either in-person at the clinic site, or remotely via video conference at the participants' convenience. From March 2020, all interviews were completed remotely to comply with the COVID-19 shelter-in-place mandate. Before starting, all participants provided consent. Each interview lasted approximately 45 min. All study procedures were approved by the UC Davis IRB (ID: 608,950).

Up to two investigators were involved in each interview (MS and/or SG). MS (he/him) a white, monolingual Assistant Professorwho was born and trained in England, and now lives and works in California, USA. Dr. Savill is a mixed-methods researcher who has led multiple projects exploring the implementation of novel interventions in psychosis from the perspective of service users, family members, behavioral health providers, and other community partners. A significant focus of Dr. Savill’s work has centered on improving pathways to care, reducing the duration of untreated illness, and improving care outcomes for people experiencing psychosis. Part of this work involves the parent study to this project evaluating the diagnostic accuracy of psychosis screening in the IBH-PC space. Dr. Savill is therefore invested in exploring the idea of screening for early psychosis in primary care spaces as a potential method to identify and support those who may benefit from specialized services. SG (she/her) identifies as an Egyptian American and White bicultural person who was born and raised in Columbus, Ohio, and has also lived in California, Texas, and Washington. Sarah has been working in mental health research for 9 years and is currently pursuing doctoral training in clinical psychology at the University of Washington researching mental health and psychosocial stressors in people of color, and sexual and gender diverse individuals. On this project, Sarah was the study coordinator of the parent study for three years and so was highly familiar with the screening process.

All interviews were conducted privately between the interviewee, MS, and/or SG.

All interviews were audio recorded and transcribed. After each batch of interviews was completed, the research team met to review field notes, discuss possible preliminary themes, and refine the interview guides. The analysis of the transcripts was conducted utilizing an inductive approach to thematic analysis [30]. Three researchers were involved in the analysis (MS, SG, JK). First, a preliminary codebook was generated by the coding team following a review of the transcripts. Each coder then independently coded the same two interviews using the codebook and then met to discuss codes and develop concordance between raters. Next, two coders (SG, JK) independently coded two interviews, after which the coding team came together to review the coding for each transcript and discuss potential modifications to the codebook. This process was repeated until all transcripts were coded. In a review of the last four interviews, no modifications to the codebook were proposed, suggesting saturation had been reached. Once the coding had been completed, the coding team worked together to combine the codes into overarching themes derived from the data. This analysis was completed using NVivo 12 [31].

## Results

In total, 12 providers and eight service users completed an interview. No participants dropped out after meeting with the research team. Participant details are presented in Table 1.

**Table 1:** Service user and provider participant demographics

Participants Demographics	N	%
Service user Participants *n*=8		
Sex Female	7	87.5
Age (mean, Std)	26.5	2.1
Race		
White^a^	5	62.5
Black or African American	1	12.5
Asian	1	12.5
More than 1 race	1	12.5
Hispanic/Latinx	2	25
PQ-B Screen		
Positive	6	75
Negative	2	25
Assessment Outcome		
FEP	2	25
Psychosis >2 years	1	12.5
CHR	2	25
No psychosis spectrum	3	37.5
Provider Demographics *n*=12		
Role		
Care Coordinator	4	33.3
Licensed Clinician	7	58.3
Senior Management	1	8.3

*Key:* *CHR* Clinical high risk, *FEP* First episode psychosis, *PQ-B* Prodromal Questionnaire – Brief

^a^Consistent with NIH reporting guidelines, this includes two individuals that identified as Hispanic/Latinx

### Acceptability of screening in a primary care setting

Regarding acceptability, two distinct domains were explored, one concerning the acceptability of universal screening in an IBH-PC setting, and the other universal screening in the GP setting.

Almost all service users and most provider participants considered the IBH department of PC as an appropriate place to screen for psychosis spectrum disorders. Provider participants suggested that the process identifies service users that might otherwise be missed, identifies individuals quicker than usual care without screening, helps initiate therapeutic conversations, supports clinical judgment, and helps identify symptoms that are difficult to assess. Some service users reported that they had not been asked such questions by health professionals previously, which then led to positive therapeutic conversations. However, some providers did express concern that the screening process increases the workload of busy care staff. Others believed the prevalence of individuals appropriate for early psychosis services in this setting would be too low to justify the work of implementing a screening program. For those unsure, evidence around how many cases such a process could expect to identify was considered critical to evaluating screening acceptability. Additionally, some providers were concerned that service user engagement was impeded when the process was completed within the session. Developing an alliance was considered particularly important for service users who had little prior experience with mental health services. Finally, depending upon the service users typically seen at each clinic, some WellSpace sites were considered more appropriate than others, where in some locations only a small percentage of the population met the 18–30 age requirement for screening and treatment eligibility.

More mixed was the idea that psychosis screening should occur during the appointment with a GP. While some service user participants were supportive, others thought that as a behavioral health concern, it was appropriate for the screening to be limited to the IBH department. Meanwhile, most providers indicated that screening in the GP clinic would not be appropriate, suggesting the same challenges and limitations evident in an IBH setting (e.g. workload, brevity of appointments) would be even more acute in that setting. Most did not consider it an appropriate use of resources (primarily time) to screen in GP clinics.

*“I mean if I'm going there for mental health and it’s mental health related questions, I think it's just further exploration in things that just get brought up and makes you think about things, which is great. Sometimes you forget those things.”*

Service user 3.

*“Right, so here at [the IBH clinic], it's probably not appropriate. I think it's most definitely not appropriate in primary care because it's going to be a step further again in terms of the diluted population.”*

Provider 9.

### Implementation of screening

**Fig. 1:**
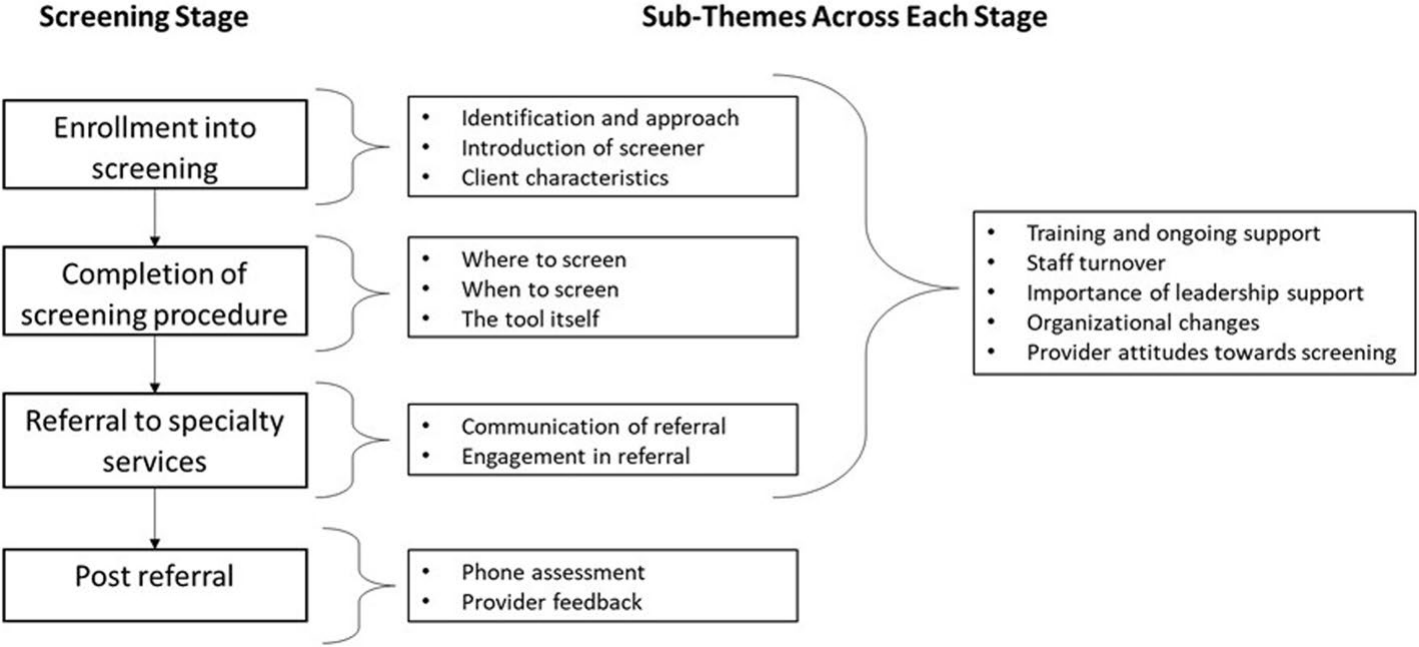
Relationship between the stages of screening, and the themes and sub-themes

Multiple factors were considered important to the successful implementation of psychosis screening in an IBH-PC setting, some related to the overall process, while others were specific to each stage of the screening process. These stages include service user enrollment into screening, the screening itself, and then the referral and post-referral stage. The relationship between the themes, sub-themes, and the different stages of the screening process are presented in Fig. 1.

### Factors impacting screening implementation overall

Factors that were considered important to the successful implementation of the process overall by providers included leadership support of screening and the ongoing training and support of all staff involved in the screening process. Factors that were considered detrimental to successful implementation included high levels of staff turnover and attempts to implement the process in the context of broader organizational change, such as changes in the intake process. Another key issue reported was provider attitudes towards the process itself. One participant talked about the importance of a respected clinician championing the process, leading others to respond positively to the program. Conversely, others talked about the challenge of implementing the screening process if individual providers were not invested in the process, or when they could not identify a direct benefit to service users.

*“They’re a pretty respected clinician. So, when they said, “Oh yeah, it's not a big deal, this is something good for the patients and it's not a big deal out of our workflow.” Then other clinicians listened to them and said, “Oh okay. Well, if [provider] can do it, then we can do it.”*

Provider 8.

### Service user enrollment in screening

A significant barrier in enrolling service users into the screening program identified by provider participants was the challenge of integrating the process into an already busy workload, particularly when service users arrived late for their appointments. Solutions proposed by providers included the importance of good teamwork between the licensed clinician and the care coordinator; asking service users to arrive early to allow plenty of time to enroll the service user before their appointment; and using checklists to both flag eligible service users as they come in, and to highlight those missed at the first appointment to prompt an approach at the subsequent session.

*Some care coordinators just had a really good working relationship with the clinician, so that helps […]. If we didn't have a care coordinator for this, I guarantee you this would have been a massive failure.*

Provider 8.

When introducing the screener, while some service users did not mind if the term “psychosis” was used, most acknowledged that others could find it challenging, particularly if it is the service users’ first behavioral health appointment. Some service users reported that if the term was used it would have either made them feel uncomfortable, or less likely to complete it. Overall, most providers indicated that they did not use the term, primarily due to concerns around stigma. Most service users and providers considered the adopted approach, which was to not typically use the term psychosis during the introduction of the screening, as most appropriate.

*“I think that would bother some people. Like, "What are you trying to say here?" I probably wouldn't recommend using that word [psychosis].”*

Service user 4.

Regarding service user characteristics, most providers suggested that service users were receptive to enrolling in the screening program, consistent with the service users interviewed. Some suggested that younger service users were particularly interested in participating. Regarding potential barriers, service users who were experiencing symptoms such as agitation or paranoia were less likely to agree to take part. Less frequently, some providers thought the stigma around mental health and psychosis also represented a barrier to participation, particularly in those who are new to behavioral health services. Finally, in some cases, concern about where the data might be going was also considered to be a barrier.

*“I feel if somebody explained to me, this is for a study at UC Davis for a mental health issue, I don't know if I would have taken the questionnaire. I would be like, "Oh, well I already am here to see this doctor, why would I want to involve other people?"”*

Service User 7.

### Tablet screening and completion stage

In the IBH-PC setting, three factors were identified as impacting the implementation of the screening. These included when the screening should take place, where, and the screening tool itself.

Depending upon the clinic, screening was either completed before the appointment or during the session itself. In almost all cases screening beforehand was considered more successful. The main barriers to screening included the typically short assessment slots allocated to each appointment, and concerns that psychosis screening breaks the therapeutic engagement by interrupting the appointment at a time when establishing good rapport is key. This was supported by some service user participants who identified the switch between the screening and then the start of the session as awkward. Receptionist staff were not considered to be appropriate individuals to introduce the screener due to their existing workload and lack of behavioral health expertise, so support staff such as behavioral health care coordinators were considered integral. Most suggested screening before, as opposed to after the session was preferable as they thought service users would be more likely to refuse post-appointment. Additionally, if service users screened positively post-session, then this would require provider time to facilitate the referral outside of the time allocated to that service user.

*“It's definitely easier to make it happen when the care coordinator's taking the lead and just doing it. Especially because if the patient arrives early, I'm with a patient, they can get it done before our clock starts ticking so to speak, on our visit. So that's nice.”*

Provider 7.

Most service users and providers thought that it was necessary for screening to take place in a private place. PC waiting rooms were described as noisy and busy, and the unusual and personal nature of the questions meant some service users felt privacy was important to answer truthfully. Regarding the screener, most service users and providers liked the questions being available electronically, considering it more ecological, secure, and easier to complete relative to paper-based surveys.

*“I'm glad I was in that little room, because I really thought like, "What the hell!?" There were some weird questions. Not confusing, just nobody's ever asked me those questions before.*

Service user 4.

### Referral and post-referral

While many participants did not report any clear obstacles to the referral procedure, facilitating service user engagement in the intake phone interview with the early psychosis specialty care clinic was considered challenging. To aid this, some providers suggested giving service users business cards with the contact details of the specialty care service so they can recognize the caller ID number, while others suggested emailing service users to provide a clearer, written explanation of the process.

In cases where the specialty care intake phone interview was completed, providers received a detailed summary of the assessment with treatment recommendations. Overall, these were not considered clinically useful. In most cases, the reports came back after the service user had already been discharged, or work with the service user had progressed to a stage where the information was redundant. Instead, most providers reported that a briefer summary with a faster turnaround would be more useful, with clear information on whether they have been accepted into care to aid their care planning efforts.

*“I wish I could be more curious in kind of, like what was going on, but really, ultimately, I just wanted to know: is the person connected or not?”*

Provider 2.

## Discussion

Overall, screening for early psychosis in an IBH-PC setting was considered feasible and acceptable both by service users and providers. Screening was seen to identify more service users faster than usual care while supporting existing clinical activities. Most providers suggested that screening in the GP consultation would not be appropriate, given concerns around even tighter time constraints, disruption to service user engagement, and the volume of non-behavioral health-related GP appointments. Participants identified multiple factors they considered important to implement early psychosis screening in an IBH-PC setting successfully, such as the importance of leadership support, onsite champions, and strong coordination between a clinician and care coordinator. Additionally, recommendations to minimize any disruption to existing services while maximizing the screening effectiveness were also proposed, such as ensuring screening took place before the appointment in a private space and utilizing electronic checklists to flag potentially eligible service users. Together, these findings aid the broader dissemination of early psychosis screening in the IBH-PC setting.

### Strengths and limitations

One strength of the study includes the fact that both service user and provider views were represented, unlike earlier studies that have examined the acceptability of psychosis screening in other settings [10]. Additionally, the cross-section of service users recruited, including those who did and did not screen positive, and those who were and were not found to be eligible for specialty care services was also significant, ensuring a broad range of screening experiences were captured. Finally, while studies examining the barriers and facilitators to early psychosis screening have been completed in CMHCs and school counseling services, to our knowledge this is the first study that has extended this to examine screening within the PC setting.

One limitation relates to the fact that all service user interviews were completed remotely via secure videoconferencing. The early interviews were conducted in this manner based on service user preference, but in the later interviews, this was due to COVID-19 and the subsequent “shelter-in-place” mandate. While the interviews themselves were successful and the remote aspect did not appear to impact participant responses, this may have created a selection bias. People who are comfortable conducting interviews remotely may be more comfortable with technology, and so more receptive to a technology-based screening program. It is also possible that participants who did not have access to computers or phones may have been excluded from the process. However, this was not raised as a barrier by any prospective participant during recruitment. Another potential limitation is that participants more critical of the program may have elected to not take part or have been more guarded in their responses. However, in at least one case a participant was vocal about participating as they saw it as an opportunity to articulate issues with the screening process, suggesting the opposite may equally be true. Additionally, while WellSpace providers were involved in the developing the interview guide and were provided a copy of the findings following analysis to provide feedback, unfortunately individuals with lived experience were not involved in designing, conducting, or reviewing the findings. In future work, actively including those with lived experience may provide further insights regarding the acceptability of incorporating the screening process into this setting. Finally, given the notable heterogeneity between primary care systems across different counties [32, 33], future work exploring the feasibility of the approach in other systems would also be informative.

### Implications

Some provider participants suggested that population-based psychosis screening identified more eligible service users faster than usual care, which is consistent with the accompanying diagnostic accuracy study [25]. Additionally, these findings are similar to those reported in a study of CMHC providers who implemented a similar screening process [10], and suggest that psychosis screening in an IBH-PC setting is appropriate and acceptable to service users and providers. However, it is notable that participants identified more challenges to implementing screening in IBH-PC, relative to CMHCs. For example, they discussed the broader case mix of service users, shorter appointment times, and being earlier in the pathway to care meaning service users may have less mental health literacy and the provider less information. To mitigate this, attention needs to be paid to implementing the process in a manner that minimizes disruption to ongoing procedures. Additionally, if the aim is to implement such procedures in IBH-PC clinics then careful consideration needs to be paid to the population the clinic typically treats, and what proportion could be eligible for local early psychosis services. For example, if the clinic primarily serves those older than 30, screening for early psychosis might not be appropriate in the US given many US-based early psychosis programs do not currently serve clients at that age [34]. In countries where the age limit for specialized early psychosis care is typically much higher (i.e., the UK [35, 36]), this may not represent such a barrier. However, it does highlight the need for further validation to ensure the screening process is still appropriate for adults up to the age of 65.

Notably, almost all providers suggested that implementing a universal psychosis screening procedure in the GP setting would be inappropriate. Most providers raised concerns about the feasibility of screening where there would be even shorter appointment times, a higher throughput of cases, and a lower prevalence of individuals presenting with psychosis spectrum disorders. However, with the push towards the integration of health and behavioral health care in the PC setting across the US [23], even if screening was limited to IBH-PC departments, such a service could be made available to an increasingly large proportion of the population.

Currently, most PC clinics in the US do not have IBH departments. The collaborative care model for depression screening and treatment within PC is gaining traction, in both PC clinics that serve commercially insured and FQHC clinics [37, 38]. While this may prompt interest in expanding to psychosis screening in PC, the results of the present study suggest providers and service users would be most amenable to psychosis screening conducted by the behavioral health staff. Future research should examine this possibility.

Lastly, the barriers and facilitators to psychosis screening implementation were found to be broadly consistent with psychosis screening in the CMHC setting, and with screening for other disorders in PC. These include the importance of regular training and support, developing an effective method of introduction, and support for computer-aided screening systems [10, 39, 40]. Interestingly, consistent with the study of psychosis screening in CMHCs [10], different factors were found to be relevant to different stages of the screening process. This suggests that to refine the implementation of such screening projects it is critical to identify where in the process challenges may be occurring (i.e. in the approach of eligible participants, screener completion, or the subsequent referral and engagement in new services), given different barriers and facilitators may be relevant to each stage. These findings are important, and likely to be relevant to screening programs beyond psychosis alone. Furthermore, these findings present a process whereby screening can be successfully implemented in this setting. This could support more deductive approaches to examining the effectiveness of screening implementation within the context of existing implementation frameworks (i.e., [41]).

## Conclusions

Screening for psychosis spectrum disorders in the PC setting has been proposed as a method to improve identification and engagement in appropriate care [17, 25]. In this study, the experiences of both service users and providers suggest that screening is acceptable in this setting, albeit with additional considerations to navigate relative to alternatives such as CMHCs. Going forward, it is critical to determine if universal screening in an IBH-PC setting leads to a significant increase in the number of eligible referrals to specialty early psychosis care, which can support prevention efforts, and potentially reduce DUP relative to individuals who navigate alternative care pathways. If successful, screening in this setting has the potential to have a significant impact on improving access to early psychosis care.

### Supplementary Information

Supplementary Material 1.

## Data Availability

The datasets used and/or analyzed during the current study are available from the corresponding author upon request. The qualitative dataset is not publicly available due to confidentiality concerns.

## Abbreviations

CMHC: Community mental health clinic
DUP: Duration of untreated psychosis
FQHC: Federally qualified health center
GP: General practitioner
IBH: Integrated behavioral health
PC: Primary care
PHQ-9: Patient heath questionnaire - 9
IRB: Institutional review board
