# Supplementary material for: Exploring the acceptability, barriers, and facilitators to psychosis screening in the integrated behavioral health primary care setting: a qualitative study
Source: BMC Health Serv Res. 2024 Aug 13;24:924. doi: 10.1186/s12913-024-11359-4 (PMC11321011; doi:10.1186/s12913-024-11359-4)
Supplement: Supplementary file 1 — Supplementary Material 1. [file 12913_2024_11359_MOESM1_ESM.zip › 12913_2024_11359_MOESM1_ESM/Wellspace Interview client v3.docx]

**Screening in Primary Care – Client Interview Guide**

**Introduction:** Thank for you agreeing to be interviewed for this study. The aim of the interview is to hear about your experiences of taking part in the screening study that started when you completed the tablet at the WellSpace clinic. We are interested to know what you felt worked well, what didn’t, and what things made it easier or harder to complete the different steps of the process.

There are no “right” or “wrong” answers. The hope is that we can learn from your experiences to understand whether it’s appropriate to conduct this type of screening in this setting, and if so, what we can do to make it a better experience for people in the future.

**Introductory question**

- Could you briefly explain what happened during the appointment when you completed the screening tablet?

**Advantages/disadvantages of the tablet-based screening tool**

- Did you like the screening tablet?
  - If so, what did you like about it?
- Is there anything you disliked about the tablet?
  - If so, what?
  - Were there any particular questions you did not like?
- Did you find it easy or difficult to use?
- Are there any changes to the tablet-based app that you would recommend?
- What did you like/dislike about it being on the tablet vs a paper copy?
- Do you think you would have been more or less likely to have completed it if it was given to you in the form of a paper questionnaire?

**Completing the screener**

- Was it clearly explained to you why you were being asked to complete the screener?
  - How was it introduced?
  - Where were you when you completed the screener (i.e. in the clinician’s office, in the waiting room)?
    - Did it feel appropriate to complete it in this location? Would you have preferred to have completed the screener in a different location?
  - Do you think the way it was introduced made you more or less likely to complete it?
  - Did completing the screener feel like a good use of your time during this appointment?
  - Could the provider have introduced the tablet in a different way that would have made you feel more comfortable about completing it?

**IF THE PROVIDER DID USE THE TERM “PSYCHOSIS” OR EQUIVALENT**

- Did the provider using the term “[psychosis]” make you feel more or less likely to complete it? Why?

**IF THE PROVIDER DID NOT USE THE TERM “PSYCHOSIS” OR EQUIVALENT**

- If the provider told you that the screener is used to identify people who may be at heightened risk for psychosis do you think you would have changed the likelihood in you agreeing to complete it? Would it have made you more or less likely to complete it?

**IF THE CLIENT SCORED ABOVE THE CLINICAL THRESHOLD**

- For how long have you been dealing with the types of experiences you reported on the tablet?
- Has any healthcare provider ever asked you about these types of experiences before?
- Did you at any point want help in dealing with these experiences? If so, did you do anything to try and get help with dealing with the experiences? What did you do?

**IF THE CLIENT SCORED ABOVE THE THRESHOLD AND DID GO ON TO COMPLETE THE PHONE ASSESSMENT**

- After completing the tablet questions, what happened next?
- Was it made clear to you why you were being referred to another service?
- How did you feel about being referred to a different service?
- Was it easy or difficult to arrange the phone contact with EDAPT?
- Did you need any kind of additional support to get in contact with EDAPT and complete the phone screen assessment?
- Can you think of anything that might have made the process easier for you?

**IF THE CLIENT SCORED ABOVE THE THRESHOLD BUT DID NOT GO ON TO COMPLETE THE PHONE ASSESSMENT**

- It appears that although you scored above the clinical cutoff on the tablet, you did not complete a phone screen assessment. Was there any reason for this?
- Was there an issue with connecting with EDAPT and starting/completing the assessment?
  - If so, is there anything that could be done differently that could make this process easier?
- Was the purpose of the referral explained clearly to you?
- Did you receive a clear explanation of what the additional services were, and how they may help you?
- Do you think that such services would be of help to you?
- Would it have been possible for you to attend services at the Sacramento EDAPT clinic?
- Was there anything else going on in your life that would have made either completing the assessment or attending the EDAPT clinic difficult?
  - If so, is there anything that could be done to make that process easier?

**IF THE CLIENT SCORED BELOW THE CLINICAL THRESHOLD**

- Given that the questions asked on the tablet did not lead to any recommended changes in care, how did you feel about completing it?
- Did it feel appropriate to complete it in this clinical setting?
- If you did later start experiencing such symptoms, would you feel comfortable reporting them on the tablet or asking your WellSpace provider for help?

**Appropriateness of the setting**

- Do you think that it was appropriate to use the tablet in a behavioral health clinic within primary care such as this?
- Do you think it would be appropriate to use the tablet during your primary care appointment?
- If the tablet was used at your primary care appointment, would you suggest any differences in how it is used?

**Final question:**

- Is there anything else about the screening and referral procedure that would be important for me to know?
- Are there any changes you would recommend we make to any part of the study to improve the patient experience?
