# Supplementary material for: Exploring the acceptability, barriers, and facilitators to psychosis screening in the integrated behavioral health primary care setting: a qualitative study
Source: BMC Health Serv Res. 2024 Aug 13;24:924. doi: 10.1186/s12913-024-11359-4 (PMC11321011; doi:10.1186/s12913-024-11359-4)
Supplement: Supplementary file 1 — Supplementary Material 1. [file 12913_2024_11359_MOESM1_ESM.zip › 12913_2024_11359_MOESM1_ESM/Wellspace Interview Provider v3.1.docx]

**Screening in Primary Care – Provider Interview Guide**

**Introduction:** Thank for you agreeing to be interviewed for this study. The aim of the interview is to understand your experiences of screening for early psychosis in this clinic using the electronic tablet. We are primarily interested in knowing what you felt worked well, what didn’t, and what things made it easier or harder to successfully implement the screening.

There are no “right” or “wrong” answers. The hope is that we can learn from your experiences to understand whether it’s appropriate to conduct this type of screening in this setting, and if so, what we can do to make it a better experience both for clients and providers in the future.

**Introductory questions:**

- What is your role here at WellSpace?
- Briefly, could you briefly describe the screening process used in your clinic?
- What is your role in the screening process?
- Does it work well? Can you identify any ways of improving the screening process at your clinic?

**Advantages/disadvantages of screening**

- What do you think are the advantages of using the screening and referral procedure?
- What do you think are the disadvantages of using the screening and referral procedure?
- What things do the clients appear to like about the screening and referral procedure?
- What things do clients appear to dislike about the screening and referral procedure?

**Comparison to previous care**

- Are you aware of clients who were referred on to early psychosis treatment via the screening tool who you think would have otherwise been missed?
- Before starting the screening study here, did you ever come into contact with a client who you suspected as having untreated psychosis. If so, what happened in terms of the care they received?
- Are you aware of clients who screened positive and were referred on for early psychosis care but who clearly did not have psychosis?

**Barriers & facilitators to implementation**

- What are the difficulties, if any, implementing the screening and referral procedure?
- Did you have any clients that refused to complete the screener? If so:
  - What happened?
  - How often did this happen?
  - Did anyone provide you with a reason?
  - In these situations, was there anything you did that addressed their concerns? Did it work? Did you ever try something that did not work?
- What is your experience of the referral procedure in the event of a positive screen?
  - Is it easy or difficult to submit the referral to the early psychosis clinic?
  - Does it seem easy or difficult for the client to go through the screening and assessment process and receive care at the early psychosis clinic?
- In the event of a positive screen, do you ever need to do additional work to support the patient/family to connect with the early psychosis program?
  - Is it usually successful?
- Were there some clients that were easier/harder to engage in the process relative to others?
  - If so, was there anything you did which addressed these issues?
  - Is there any way that the screening process could be modified to better support these clients?
- Have you had any clients that refused a referral in the event of a positive screen? If so:
  - What happened?
  - How often did this happen?
  - Did anyone ever provide you with a reason?
  - In these situations, was there anything you did that was able to address their concerns? Did it work? Did you ever try?
- Is it challenging to add the screening and referral procedure to your ongoing work commitments?
  - If so, are there any changes you can think of that would make the process easier to accommodate?
  - Have there been any changes that have already occurred that have made it easier?
- Do you feel adequately prepared to explain the screening and referral procedure to clients?

**Leadership support**

- Do you feel that the leadership here at WellSpace have been actively supportive of the screening study?

**If so**,

- - what things have leadership done to be supportive?
  - Have these things had an impact of the implementation of the screening and referral procedure? Have they made it easier or harder?

**If not**,

Do you think this has impacted the implementation of the screening study? If so, how?

- Did you feel adequately supported by the research staff?

**If so,**

- - What sort of things have the research staff done to make you think this is the case?
  - Have these things had an impact of the implementation of the screening and referral procedure? Has it made it easier or harder?

**If not,**

- - Do you think this has impacted the implementation of the screening study at all? If so, how?
- Are there additional supports that could help?

**Using the tablet-based screening tool**

- What is your experience of using the tablet-based app?
  - Did you like it?
  - Did you find it easy or difficult to use?
  - Are there any changes to the tablet-based app that you would recommend?
  - Was the training helpful or unhelpful?
- How do the clients typically respond when they were handed the tablet?
  - Do you ever receive any specific feedback from clients about the tablet?
  - Do clients typically find it easy or difficult to use?
  - Do you think it being tablet- as opposed to paper- based impacts the likelihood of clients agreeing to complete the screener? Do you think it makes it more or less likely?
  - What did you like/dislike about is being on the tablet vs a paper copy?

**Final questions:**

- Based on your experiences, do you think it is appropriate to screen for early psychosis in a behavioral health setting within primary care such as this? If so, why/ why not?
- What do you think about screening for early psychosis directly in primary care, as opposed to only giving the tablet to clients that are referred to your integrated behavioral health service?
- What changes would you recommend to improve the screening and referral procedure (if any)?
